# Isolation and Molecular Characterization of 1-Aminocyclopropane-1-carboxylic Acid Synthase Genes in *Hevea brasiliensis*

**DOI:** 10.3390/ijms16024136

**Published:** 2015-02-16

**Authors:** Jia-Hong Zhu, Jing Xu, Wen-Jun Chang, Zhi-Li Zhang

**Affiliations:** 1 Key Laboratory of Tropical Crop Biotechnology, Ministry of Agriculture, Institute of Tropical Bioscience and Biotechnology, Chinese Academy of Tropical Agricultural Sciences, Haikou 571101, China; E-Mails: hjzhu1010@163.com (J.-H.Z.); changwenjun@itbb.org.cn (W.-J.C.); 2 Hainan Academy of Agricultural Sciences, Haikou 571100, China; E-Mail: xujing6732807@126.com (J.X.)

**Keywords:** *Hevea brasiliensis*, 1-aminocyclopropane-1-carboxylic acid synthase, gene expression, ethylene

## Abstract

Ethylene is an important factor that stimulates *Hevea brasiliensis* to produce natural rubber. 1-Aminocyclopropane-1-carboxylic acid synthase (ACS) is a rate-limiting enzyme in ethylene biosynthesis. However, knowledge of the *ACS* gene family of *H. brasiliensis* is limited. In this study, nine *ACS*-like genes were identified in *H. brasiliensis*. Sequence and phylogenetic analysis results confirmed that seven isozymes (HbACS1–7) of these nine *ACS*-like genes were similar to ACS isozymes with ACS activity in other plants. Expression analysis results showed that seven *ACS* genes were differentially expressed in roots, barks, flowers, and leaves of *H. brasiliensis*. However, no or low *ACS* gene expression was detected in the latex of *H. brasiliensis*. Moreover, seven genes were differentially up-regulated by ethylene treatment.These results provided relevant information to help determine the functions of the *ACS* gene in *H. brasiliensis*, particularly the functions in regulating ethylene stimulation of latex production.

## 1. Introduction

As a gaseous phytohormone, ethylene is produced in most plant tissues and play an important role in regulating plant growth and developmental processes, including seed germination, root initiation, root gravitropism, fruit ripening, flower and leaf senescence, abscission, and stress responses [1,2]. In plants, ethylene is synthesized from *S*-adenosyl-l-methionine (SAM) via 1-aminocyclopropane-1-carboxylic acid (ACC). The conversion of SAM to ACC by ACC synthase (ACS) and the conversion of ACC to ethylene by ACC oxidase (ACO) are two key steps in ethylene biosynthesis [3,4]. In general, the rate-limiting step in ethylene biosynthesis involves the conversion of SAM to ACC, and an increase in ethylene production is associated with a rapid increase in cellular ACS activity [5,6]. ACSs are encoded by a multi-gene family, whose members are differentially regulated by plant developmental, environmental, and hormonal signals [7,8,9]. The first *ACS* gene was cloned from zucchini [10]; Since then, researchers have isolated and extensively studied numerous *ACS* genes from various plant species, such as *Arabidopsis*, *Dianthus caryophyllus*, *Solanum lycopersicum*, *Prunus salicina*, and *Pyrus pyrifolia* [2,7,11,12,13]; However, the *ACS* genes in rubber tree (*Hevea brasiliensis*) have not yet been investigated.

Rubber tree is an important industrial crop for natural rubber (*cis*-polyisoprene) production. *cis*-polyisoprene is synthesized in highly specialized cells called laticifers that are differentiated from the cambium and arranged in rings. The cytoplasmic contents of laticifers are expelled in the form of latex when the bark is wounded or tapped [14,15,16]. Ethrel (ethylene releaser) is widely applied on the bark of rubber trees to stimulate latex production by improving latex flow and latex regeneration [14]. Rubber production depends on exogenous ethylene (applied as ethrel); This process also relies on an increase in the endogenous ethylene production of rubber tree by exogenous ethylene treatment; however, the molecular mechanisms of exogenous ethylene action on rubber tree remain poorly understood [17,18,19]. Relative ethylene biosynthesis and signaling genes have been identified in *H. brasiliensis* [19,20,21]. Three members of the multigene family encoding ACC oxidases in *H. brasiliensis* were isolated, of which *HbACO2* and *HbACO3* showed induced expression in responses to ethylene stimulation [20]. ACS is a rate-limiting enzyme in the ethylene biosynthesis pathway. Determination of the effect of ethylene treatment on ACS activity and gene expressions are useful to further reveal the molecular mechanisms underlying ethylene stimulation of latex production. However, thus far, few reports have been published regarding ACS in rubber tree [20,21]. High-throughput sequencing data of *H. brasiliensis* have been obtained, and this availability has provided researchers with opportunities to study *Hevea ACS* genes [22,23,24,25,26]. In this study, the gene structure, phylogenetic characteristics, and expression patterns of *Hevea ACS* genes were identified and described. The functions of *Hevea ACS* genes in ethylene stimulation of latex production were also discussed. The results of this study provided useful information for future studies on the structure and function of *ACS* genes in regulating ethylene stimulation of latex production and other important biological processes. This study could also provide the basis to identify and characterize *ACS* genes in other species.

## 2. Results

### 2.1. Cloning, Identification, and Structure Analysis of the HbACS Gene Family

**Figure 1:**
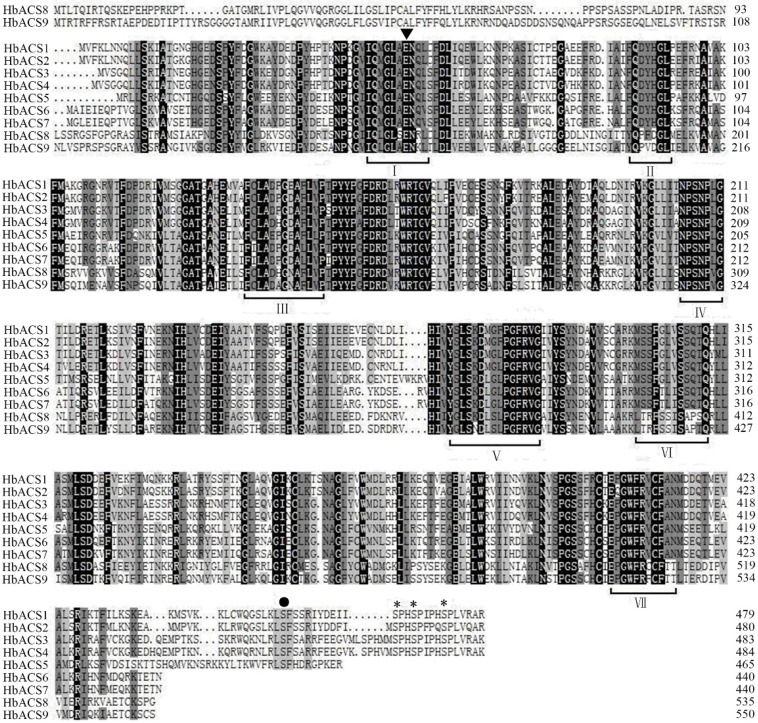
Amino acid sequence alignment of *HbACS* genes. The conserved glutamate residue (E) marked with an arrowhead is involved in substrate specificity. The seven highly conserved regions (I–VII) among all ACC synthases are underlined. Ser residues implicated in calcium-dependent protein kinase (CDPK) and mitogen-activated protein kinase (MPK6) phosphorylation are marked by a black dot and asterisks, respectively.

To identify the potential members of the *ACS* gene family in the rubber tree, we used all of the *Arabidopsis ACS* genes as queries and obtained all possible *ACS* genes by searching the NCBI database. A total of nine *ACS*-like genes labeled as *HbACS1–9* were assembled from the rubber tree on the basis of the BLASTP search. The full-length cDNAs and DNAs of the nine *ACS*-like genes were cloned and identified by PCR amplification and sequencing. The deduced proteins of the *ACS*-like genes contained approximately 440 to 550 amino acids (predicted molecular mass = 49.18 to 60.48 kDa) with isoelectric points ranging from 5.58 to 8.23 (Table 1). The deduced ACS-like proteins showed close identities to ACS from other plants and exhibited the typical structure of plant ACS. All of the ACS-like isozymes contained the seven conserved boxes found in ACS from *Arabidopsis*, tomato, and other plant species; ACS-like isozymes also contained a conserved glutamate residue involved in substrate specificity. Four ACS isozymes (HbACS1–4) contained four Ser residues that function as targets of calcium-dependent protein kinase (CDPK) and mitogen-activated protein kinase (MAPK) phosphorylation; By contrast, HbACS5 only contained the CDPK target motif (Figure 1). Sequence alignment results indicated that HbACS1–7 showed more than 50% amino acid identity between two with the maximum percentage of nucleotide and amino acid sequence identities found between HbACS6 and HbACS7 (93.8% and 93.1%, respectively). However, HbACS8 and HbACS9 showed <40% identity with the seven other HbACS isozymes (HbACS1–7) (Table S1).

**Table 1:** Information of *HbACS*-like genes and their predicted proteins.

Gene	GenBank Accession No.	ORF (bp)	Predicted Protein			
			Size (aa)	Type	*M*w (kDa)	pI
*HbACS1*	KJ911898	1443	480	Type 1	54.05	6.35
*HbACS2*	KJ911899	1446	481	Type 1	54.43	5.70
*HbACS3*	KJ911900	1452	483	Type 1	54.20	5.74
*HbACS4*	KJ911901	1455	484	Type 1	54.60	7.24
*HbACS5*	KJ911902	1398	465	Type 2	52.66	8.94
*HbACS6*	KJ911903	1323	440	Type 3	49.18	5.69
*HbACS7*	KJ911904	1323	440	Type 3	49.25	5.58
*HbACS8*	KJ911905	1608	535	Putative AAT	59.24	8.23
*HbACS9*	KJ911906	1653	550	Putative AAT	60.48	6.23

### 2.2. Phylogenetic Analysis

**Figure 2:**
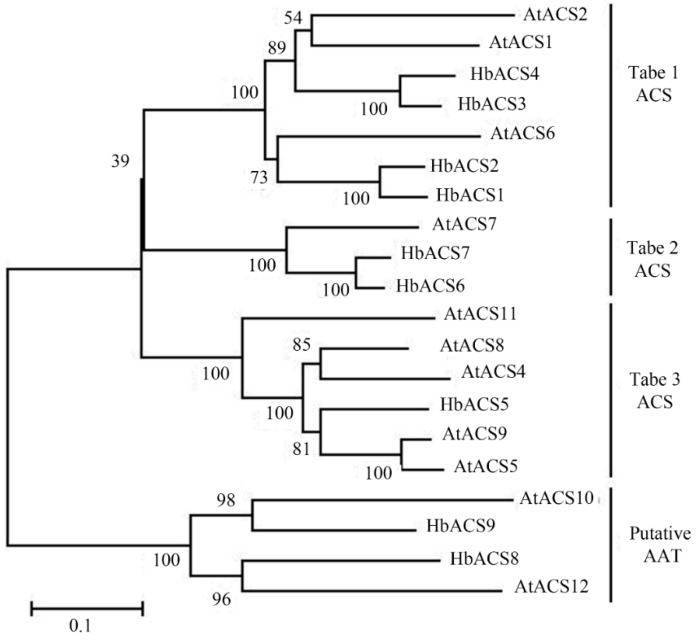
Phylogenetic analysis of *Hevea* and *Arabidopsis* 1-Aminocyclopropane-1-carboxylic acid synthase (ACS)-like protein sequences. The accession numbers of *Arabidopsis* ACS-like known proteins in GenBank are listed as follows: AtACS1(NP_191710); AtACS2(Q06402); AtACS4(NP_179866); AtACS5(AAG50098); At-ACS6(T13019); At-ACS7 (AAG48754); AtACS8(AAG50090); AtACS9(AAG48755); AtACS10 (NP_564804); AtACS11(NP_567330); and AtACS12(NP_199982).

ACS proteins can be divided into three types (types 1, 2 and 3) based on the amino acid sequence of the *C*-terminal region [7]. Phylogenetic and molecular evolutionary analyses were conducted using MEGA version 6 [27] by comparing nine ACS-like sequences from the rubber tree with 11 ACS-like sequences from *Arabidopsis* (Figure 2). The results indicated that seven HbACS-like peptides were similar to eight AtACS peptides that are involved in the synthesis of ACC [7], in which HbACS1–4, HbACS5, and HbACS6–7 were classified as types 1, 2 and 3, respectively. HbACS 8 and 9 were closely matched to AtACS10 and AtACS12, which are presumed as amino acid transferases without ACS activity [7].

### 2.3. Intron and Exon Organization of HbACS Genes

**Figure 3:**
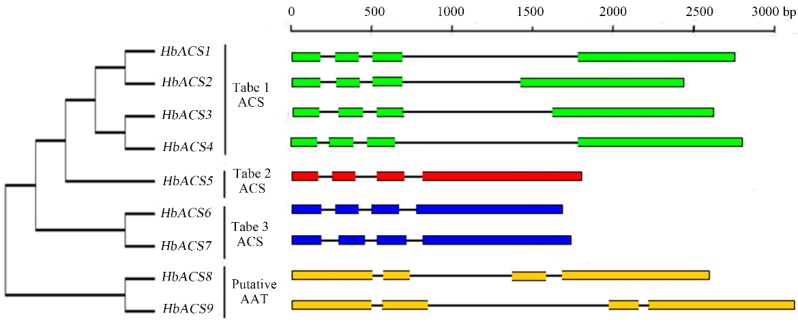
Neighbor-joining phylogenetic tree and intron-exon structures. The phylogenetic tree (part of the left side) was constructed from HbACSs using the MEGA 6.0 program with the NJ method. Intron and exon structural organization of *HbACS* genes are described on the right side. Introns and exons are represented by black lines and colored boxes, respectively.

In general, two, three, or four introns are possibly present in the genomic sequences of *ACS*, but the position of each intron is conserved [28]. The genomic structures of *HbACS* genes were analyzed by comparing the corresponding cDNA sequences with the PCR fragments amplified from genomic DNA. The results showed that all of the *HbACS* genes contained four exons and three introns (Figure 3). Although introns differ in length, these non-coding regions were located at a similar position in the 5'-end of a gene, as reported in other plants; these introns were also typically flanked by GT and AG boundaries. In addition, different *HbACS* genes in each type exhibited similar exon/intron structure, which could provide additional evidence to support the phylogenetic relations in a particular gene family. For instance, four genes (*HbACS1–4*) clustered in type 1 shared the same gene structure, including the patterns of the positions and lengths of introns; However, these genes were different from those under a different type.

**Table 2:** The putative *cis*-elements in the promoters of *HbACS* genes.

Gene	Size	Hormone Response Element	Stress Response Element	Other Element
*HbASC1*	823	CGTCA-motif, TATC-box	MBS	GCN4_motif, Skn-1_motif
*HbASC2*	1484	CGTCA-motif, ERE GARE-motif, TATC-box TGA-element	MBS HSE TC-rich repeats	O_2_-site, Skn-1_motif, as-2-box
*HbASC3*	1625	ABRE, CGTCA-motif, ERE, TCA-element	AT-rich element, HSE	GCN4_motif, Skn-1_motif
*HbASC4*	1553	ABRE, P-box, TGA-element	ARE, HSE, LTR, MBS, TC-rich repeats	Skn-1_motif, as-2-box, circadian
*HbASC5*	1575	ABRE, AuxRR-core, GARE-motif	ARE, Box-W1, C-repeat/DRE, HSE, MBS, TC-rich repeats, W box	GCN4_motif, Skn-1_motif
*HbASC6*	1110	GARE-motif, TGA-element	Box-W1, HSE, MBS, TC-rich repeats, W box	O_2_-site, Skn-1_motif^2^
*HbASC7*	2036	P-box, AuxRR-core, GARE-motif, TCA-element	ARE, HSE, MBS, TC-rich repeats	CAT-box, O_2_-site, RY-element, Skn-1_motif, circadian

ABRE, abscisic acid-responsive element; ERE, ethylene-responsive element; ARE, element essential for the anaerobic induction; HSE, heat stress responsiveness; LTR, low-temperature responsiveness; MBS, MYB binding site involved in drought inducibility; TCA-element, salicylic acid-responsive element; TC-rich repeats, defense and stress responsiveness; CGTCA-motif, MeJA responsiveness; GARE-motif, gibberellin-responsive element; Circadian, circadian control; GCN4_motif, endosperm expression; Skn-1_motif, endosperm expression; Box-W1/W box, funga elicitor-responsive element; TATC-box, gibberellin-responsive element; WUN-motif, wound-responsive element; TGA-element, auxin-responsive element; P-box, gibberellin-responsive element; AuxRR-core, auxin-responsive element; O_2_-site, zein metabolism regulation; CAT-box, meristem expression; RY-element, seed-specific regulation; C-repeat/DRE, cold- and dehydration-responsiveness; as-2-box, shoot-specific expression and light responsiveness

### 2.4. Cis-Elements of HbACS Promoters

To further understand the transcriptional regulation and potential functions of HbACS genes, the upstream promoter sequences of *HbACSs* (800–2000 bp upstream of the initiation codon) were isolated and predicted (Figures S1–S7). A number of *cis*-elements involved in hormone response stress response and tissue-specific were found in the upstream sequences of these *HbACSs* (Table 2 and Figures S1–S7). All *HbACS* promoters contained two or more *cis*-elements involved in plant hormone response, such as the abscisic acid response element, ethylene-responsive element, gibberellin responsive element, and MeJA-responsive element. All *HbACS* genes contained one or more of the *cis*-elements involved in stress response, such as low-temperature responsive, heat stress responsive, wound-responsive, and anaerobic response element. Moreover, the cell and tissue-type expression CAT-box, GCN4_motif and Skn-1_motif were also found in the upstream sequences of these *HbACSs* (Figures S1–S7). *Cis*-elements in the *HbACS* promoter regions might be essential in mediating responses to hormone and stress response as well as in growth and development.

### 2.5. Expression Analysis of HbACS Genes in Hevea Tissues

**Figure 4:**
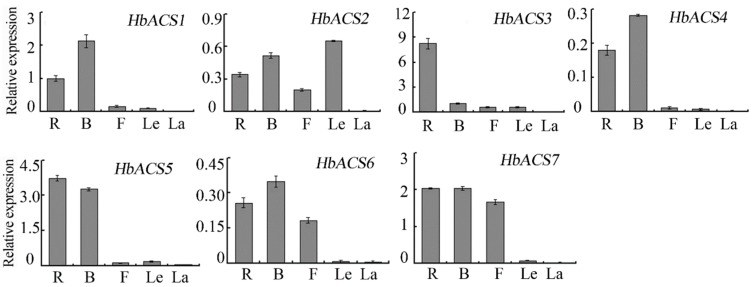
*HbACS* gene expression in various tissues, *i.e*., roots (R), barks (B), flowers, leaves (Le) and latex (La). Data are means ± standard error calculated from three independent biological replicates.

To understand the potential functions of specific ACS isozymes in *Hevea*, we analyzed the tissue-specific expression pattern of *HbACS* genes in different *Hevea* tissues. Our results revealed that all of the seven *HbACS* genes were differentially expressed in roots, barks, flowers, and leaves. We also compared the transcripts of *HbACS* genes in each tissue and found the following predominant *HbACS* gene expressed in various tissues: *HbACS1* and *HbACS4* in barks; *HbACS2* in leaves; *HbACS3* and *HbACS5* in roots. The expression levels of *HbACS6* and *HbACS7* were not significantly different in roots, barks, and flowers; the expressions of these two genes were almost undetectable in leaves. However, these genes were expressed at very low levels or not expressed in the latex (Figure 4).

**Figure 5:**
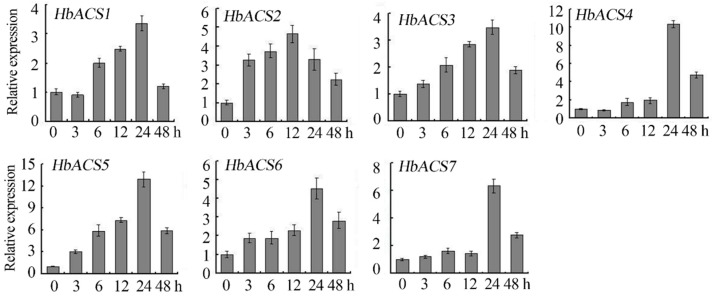
Expression of the *HbACS* genes in the bark after ethrel was applied. Data are means ± standard error calculated from three independent biological replicates.

### 2.6. Expression Analyses of HbACS Genes in Response to Ethrel Treatment

Ethrel has been regularly applied on the bark of rubber tree to stimulate latex production. To study the potential functions of *HbACS* genes in the ethylene stimulation of latex production, we analyzed the *HbACS* transcript levels in the latex and barks after the trees were treated with ethrel. The results showed that the expressions of seven ACS genes in the barks were up-regulated by at least threefold after the trees were treated; the maximum expression level was reached 24 h after stimulation except the expression of *HbACS2*, which reached the maximum level 12 h after stimulation (Figure 5). However, the transcripts of these ACS genes found in the latex after ethylene treatment were very low or undetectable.

## 3. Discussion

Multi-gene families encoding ACS have been cloned and characterized from various plant species. Genome screening results have revealed the existence of 12 *ACS* genes in *Arabidopsis*, 6 *ACS* genes in rice, 10 *ACS* genes in grapevine, and 11 *ACS* genes in poplar [29]. Thus far, *ACS* genes from the rubber tree have not been completely cloned and investigated, although these genes may be implicated in regulating the development and latex regeneration of *Hevea* [19,20,21]. In this study, at least nine ACS-like genes were found in rubber tree; Sequence and phylogenetic analysis results showed that seven isozymes of these ACS-like genes (*HbACS1–7*) were similar to the eight AtACS isozymes with ACS activity [7].

The post-translational regulation of ACS proteins by phosphorylation has been identified in *Arabidopsis*, tomato, and other plants; this process is important in regulating the stability of ACSs [5,29]. ACS proteins are classified into three categories based on the absence or presence of *C*-terminal phosphorylation motifs [30]. HbACS1–4 belong to type 1 ACS isozymes and contain a Ser residue for CDPK phosphorylation and a long *C*-terminal tail with the three conserved Ser residues for MAPK phosphorylation [5,31]. LeACS2, a tomato type 1 ACS, is phosphorylated by CDPK and MAPK in response to a wounding signal, resulting in increased ACS activity and ACC content [31]. Similar to other type 2 ACS isozymes, HbACS5 only carries the motif for CDPK phosphorylation. The function of CDPK phosphorylation may also be implicated in regulating protein stability and ethylene synthesis [32]. Type 3 isozymes, particularly HbACS6 and HbACS7, carry neither of the target sites required in the *C*-terminal tail for protein phosphorylation. Recently, AtACS7, a type 3 ACS, was found to be phosphorylated at Ser216, Thr296, and Ser299 by AtCDPK16 *in vitro*, which is different from the previous expectation that type 3 ACS proteins have no predicted CDPK phosphorylation sites and may not be a substrate of CDPKs [2]. Whether HbACS6 and HbACS7 are phosphorylated by CDPK needs further study.

The members of the *ACS* multi-gene family exhibit tissue expression patterns that vary among several plant species. Among the nine *ACS* genes in the banana genome, only *MAACS1* is expressed during banana fruit ripening [6]. The *ACS* genes (*DCACS2* and *DCACS3*) of carnation are preferentially expressed in the style; by comparison, the mRNA of *DCACS1* is most abundant in the petals [33]. Our results similarly showed that *HbACS* genes had tissue-specific expression patterns and were differentially expressed in roots, barks, flowers, and leaves of *H. brasiliensis*. Furthermore, these genes were almost undetectable in the latex. Moreover, the transcripts of the three members of the ACO gene family involved in ethylene synthesis were also undetectable in the latex [20]; this result indicated that fully differentiated latex cells may not express *ACS* and *ACO* genes and cannot synthesize endogenous ethylene.

Ethylene influences plant flower opening, fruit ripening, senescence, root gravitropism, and abscission [1,2,3,4,34]. Ethylene can promote endogenous ethylene production via an auto-activating mechanism that regulates ethylene biosynthetic genes [35]. An increase in the transcript accumulation of *LEACS2* induced by ethylene treatment can promote ethylene auto-activation and result in respiratory climacteric [11,31]. Exogenously applied ethylene has been found to induce autocatalytic ethylene production in petals of *D. superbus* var. *longicalycinus* with simultaneous accumulation of transcripts of *DsuACS1* [13]. Ethylene stimulation can increase latex production in rubber tree, and yield stimulation depends on exogenous ethylene and increased endogenous ethylene in the barks [19]. In this study, the seven *HbACS* genes found in the bark of rubber tree were differentially up-regulated by exogenous ethylene. However, no detectable transcripts of the *ACS* genes were found in latex after ethylene stimulation was performed. Similar results were found for *ACO* genes in rubber tree: no expression of *ACO* genes was detected in latex; the transcriptions of *HbACO2* and *HbACO3* in the barks were induced in response to ethylene [20]. Therefore, autocatalytic ethylene production in the bark of the rubber tree via the upregulation of biosynthetic genes by ethylene may be related to the stimulation of latex production. However, gene expression levels of *ACS* genes not always reflect actual protein levels, and that post-translational modifications such as phosphorylation also play an important role in protein stability and activity. In addition, as is a gaseous molecule, ethylene can freely diffuse from one cell to a neighboring cell, evoking mainly local responses [36]. Thus, our hypothesis requires further studies on ethylene content and ethylene biosynthetic enzyme activity in both the barks and latex.

In addition to developmental and hormone signals, ACS genes were also regulated by various abiotic and biotic stresses, including wounding, freezing, drought, anaerobic conditions, fungal elicitor, and salt stress [7,8,9]. In this study, we found that that the promoter regions of *HbACSs* contained several abiotic/biotic stress response elements, such as low temperature, heat, wound, anaerobic conditions, and fungal elicitor (Table 2). These observations suggest that *HbACSs* may be involved in responding to several abiotic and biotic stresses. Although *cis*-regulatory elements play important roles in regulating gene expression, further studies are needed to verify the relationship between the expression profiles of *HbACSs* and the *cis*-regulatory elements in their promoter regions.

## 4. Materials and Methods

### 4.1. Plant Materials and Treatments

Rubber trees (*H. brasiliensis* clone 7-33-97) were planted in the experimental farm of the Chinese Academy of Tropical Agricultural Sciences in Hainan Island in China. Six-year-old virgin trees (untapped trees) were treated with 1% ethrel in accordance with the method of Hao and Wu [15]. Latex and bark samples were collected at 0, 3, 6, 12, 24 and 48 h after treatment. The latex was allowed to drop directly into liquid nitrogen in an ice kettle. The frozen latex powder was then stored at −70 °C or used immediately to extract RNA. Fresh leaves, flowers, and barks were immediately ground to form powder in liquid nitrogen and stored at −70 °C or immediately used to extract nucleic acid.

### 4.2. Nucleic Acid Extraction and cDNA Synthesis

Genomic DNA was isolated from the leaves in accordance with the method described by Allen *et al*. [37]. Total RNA was extracted from the latex [38] and from other tissues [39]. First-strand cDNA was synthesized from the total RNA by using a PrimeScript RT reagent kit (Takara Biotechnology, Dalian, China) according to the manufacturer’s instructions.

### 4.3. Cloning and Identification of ACS Genes

To identify the *ACS* homologs in *H. brasiliensis*, we used *Arabidopsis ACS* genes (*AtACS1–AtACS11*) as queries and searched for whole-genome contigs (WGS) of *H. brasiliensis*. The contigs of putative *ACS* genes were then assembled. On the basis of the assembled sequences, we designed and used multiple pairs of primers (Table S2) to amplify the cDNA and genomic DNA of putative *ACS* genes. The products were then cloned in the pMD18-T cloning vector (TaKaRa Biotechnology, Dalian, China). Afterward, their sequences were analyzed in GenBank by using the BLAST program. Multiple amino acid sequence alignment and phylogenetic tree analysis were performed using the MEGA 6.0 software. The gene structure schematic of *HbACSs* was drawn using the web server GSDS (http://gsds.cbi.pku.edu.cn/). The isoelectric point (pI) of HbACS was predicted using the Compute pI/Mw software (http://www.expasy.ch/tools/pi_tool.html).

### 4.4. Promoter Region Analysis of HbACS Genes

To investigate the *cis*-elements in promoter sequences of *HbACS* genes, genomic DNA sequences located at 800–2000 bp upstream of the initiation codon (ATG) for all *ACS* genes were obtained from WGS of *H. brasiliensis* (GenBank:AJJZ01000000) [26]. The results were further confirmed by sequencing the PCR products with genomic DNA as templates, and primer pairs used were given in Table S2. The PLACE database (http://www.dna.affrc.go.jp/PLACE/) was used to analyze the *cis*-elements in the promoter regions.

### 4.5. Expression Analysis

Quantitative real-time PCR (qRT-PCR) was performed on Mx3005P Real-Time PCR System, following the manufacturer’s protocol, using the Reagent kit for SYBR Green analysis (TransGen Biotech, Beijing, China) and specific primers (Table S2). Yellow leaf-specific protein gene (*YLS8*), as a mitosis protein gene, was considered a suitable reference for qRT-PCR analyses in *H. brasiliensis* and *A. thaliana* [40,41,42]. Thus, *YLS8* was used as an endogenous reference gene for cDNA normalization. Primer efficiency was verified by the presence of single PCR amplicons on agarose gel electrophoresis. qRT-PCR conditions were as follows: 30 s at 95 °C for denaturation, 40 cycles for 10 s at 94 °C, 20 s at 60 °C, and 15 s at 72 °C for amplification. The data obtained from the qRT-PCR were clustered according to the instructions provided by Stratagene (Santa Clara, CA, USA). All of the relative expression data were based on three individual reactions.

## 5. Conclusions

In this study, seven *HbACS* genes were cloned and characterized, the expression profiles were investigated, and their possible functions were discussed. The results show that *HbACS* genes were differentially expressed in roots, barks, flowers, and leaves except in latex. *HbACS* genes were also differentially up-regulated by ethylene in barks, which may be related to ethylene stimulation of latex production, but this hypothesis requires further studies.

## Supplementary Materials

Supplementary materials can be found at http://www.mdpi.com/1422-0067/16/02/4136/s1.
